# Functional Evaluation Using Inertial Measurement of Back School Therapy in Lower Back Pain

**DOI:** 10.3390/s20020531

**Published:** 2020-01-18

**Authors:** Claudia Celletti, Roberta Mollica, Cristina Ferrario, Manuela Galli, Filippo Camerota

**Affiliations:** 1 Physical Medicine and Rehabilitation, Umberto I University Hospital, 00161 Rome, Italy; clacelletti@gmail.com (C.C.); roberta.mollica@uniroma1.it (R.M.); 2 Department of Mechanic, Politecnico di Milano, 20124 Milan, Italy; cristina.ferrario@polimi.it; 3 Department of Electronics, Information and Bioengineering (DEIB) Politecnico di Milano, 20133 Milan, Italy; manuela.galli@polimi.it

**Keywords:** back school, inertial sensor, lower back pain, rehabilitation, stability, timed up and go test

## Abstract

Lower back pain is an extremely common health problem and globally causes more disability than any other condition. Among other rehabilitation approaches, back schools are interventions comprising both an educational component and exercises. Normally, the main outcome evaluated is pain reduction. The aim of this study was to evaluate not only the efficacy of back school therapy in reducing pain, but also the functional improvement. Patients with lower back pain were clinically and functionally evaluated; in particular, the timed “up and go” test with inertial movement sensor was studied before and after back school therapy. Forty-four patients completed the program, and the results showed not only a reduction of pain, but also an improvement in several parameters of the timed up and go test, especially in temporal parameters (namely duration and velocity). The application of the inertial sensor measurement in evaluating functional aspects seems to be useful and promising in assessing the aspects that are not strictly correlated to the specific pathology, as well as in rehabilitation management.

## 1. Introduction

Lower back Pain (LBP) is a well described and extremely widespread health problem [1]. LBP is a pain that goes from the twelfth rib to the lower gluteal folds; pain can also spread to the lower limbs for one day or more [1]. This condition is the main cause of absence from work and activity limitations in much of the world. The consequence is a heavy economic burden for subjects, families, communities, industry, and governments [2]. Of the 291 conditions studied in the 2010 Global Burden of Disease (GBD) report, LBP had the highest load. LBP is the leading cause of disability globally [3].

The main components to treat this condition are education, reassurance, analgesic drugs, and non-pharmacological therapies. During the treatment, periodic check-ups are recommended based on individual patient needs, such as prognosis, treatment prescribed, and remaining concerns about serious pathological abnormality [4].

Chronic LBP is defined as lower back pain that lasts for over 12 weeks. Generally, one-third of the patients with LBP reported that in the year after an acute episode, lower back pain was of moderate intensity [2]. In patients with chronic back pain, a multidisciplinary approach leads to better results when combined with medical, rehabilitative, and psychological treatments [5].

Among other rehabilitation approaches, back schools (BS) are interventions that comprise an education component and exercises. BS are training programs with lessons given by a therapist to patients or workers, with the aim of treating or preventing lower back pain [6]. Several studies have demonstrated the efficacy of BS in reducing and managing lower back pain [7]. BS, due to the validity of their educational exercises, enhance the quality of life, reduce disability induced by LBP [8,9], and also improve mental well-being.

The aim of this study is to evaluate not only the efficacy of BS therapy in reducing pain but also in functional improvement, an aspect strictly related to pain but normally not evaluated in the studies that focus on assessing pain relief. A new and simple gait evaluation method is used to make the analysis. In particular, stability and ability to perform functional tests, such as the timed “up and go” test, are evaluated in order to verify if a rehabilitation program based on BS therapy is able to improve stability and walking.

## 2. Materials and Methods

Patients were recruited from the Rehabilitation Ambulatory Service of Umberto I University Hospital. All participants signed informed consent forms after receiving detailed information about the study’s aims and procedures for the Declaration of Helsinki.

### 2.1. Eligibility Criteria

Patients were included in the study if they had lower back pain that had lasted for more than six weeks that was associated with limitations of motion. The presence of vertebral infections; tumoral metastasis; fractures and neoplasm; rheumatological, neurological, or oncological disease; previous back surgery; severe cognitive impairments; or pregnancy was considered an exclusion criterion.

### 2.2. Intervention

The BS program was supervised by a multidisciplinary professional team. A total of 10 one-hour sessions scheduled 3 times a week were carried out. The adopted rehabilitation program was chosen by considering the effectiveness of the BS on LBP reported in previous studies. The details of the program followed in this study are described below.

The first treatment session was used to provide subjects with basic anatomical knowledge of the spine and its functions; the correct ergonomic positions to be maintained in everyday life were also shown. During the following 9 sessions, the physiotherapists supervised the activities, which consisted of exercises based on diaphragmatic breathing (10 min), self-stretching of the trunk muscles (10 min), strengthening of erector muscles of the spine, abdominal strengthening, and postural exercises. The tasks were divided into 3 sets of 10 repetitions for each one; 3 min of rest was provided between each series. Explanations of the ergonomic position of the spine and how to introduce self-correction in daily life were provided for the whole duration of the treatment.

### 2.3. Health State: Clinical Evaluations

Patients were evaluated before and after physiotherapy treatment with the following clinical scales:

1. The *numeric rating scale* (NRS) is a rapidly administered 11-point numeric scale used to roughly measure any kind of pain, with a score ranging from 0 (no pain) to 10 (acute pain) [10];
2. The *Oswestry disability index* (ODI), also known as the Oswestry lower back pain disability questionnaire, is considered the “gold standard” of lower back functional outcome tools and consists of 10 sections, with a score varying from 0 to 5 for each one. A low score indicates minimal disability; the disability is more severe for higher scores [11];
3. The *performance-oriented mobility assessment* (POMA) scale was developed by Tinetti in 1986 to assess the mobility and risk of falling of the elderly [12]. This scale was chosen because it is very reliable and widely used. In this study, we used the balance scale of the POMA, which evaluates the positions and changes in position of the subject, assessing stability tasks. Each item is scored on a two- or three-point scale, where the maximum is 18 [13];
4. The *timed up and go test* (TUG) is a clinical test that evaluates the balance and mobility of a subject [14,15]. In the traditional TUG test, a stopwatch is used to measure how long it takes a subject to lift off a chair, walk 3 m, turn 180°, return to the chair, and sit back down.

### 2.4. Biomechanical Evaluation

#### Instrumentation

**Figure 1:**
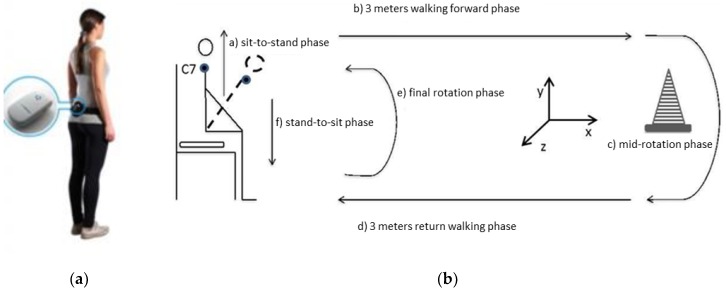
(**a**) Inertial measurement unit (IMU) position and (**b**) timed up and go test (TUG) phases.

In this study, we evaluated the TUG as both a time test and also using an inertial measurement unit (IMU). The commercial name of the device used is a G-Sensor instrument (BTS SpA, Milan, Italy). The communication with the receiving unit (personal computer) takes place via a Bluetooth connection. The associated software (BTS^®^ G-Studio) is used to acquire, process, and archive data. In the IMU there is a triaxial accelerometer (16 bits/axes, up to 1000 Hz) with different sensitivities (±2, ±4, ±8, ±16 g), a triaxial 16-bit magnetometer (±1200 μT, up to 100 Hz), and a triaxial gyroscope (16 bits/axes, up to 8000 Hz) with multiple sensitivities (±250, ±500, ±1000, ±2000°/s). The G-Sensor is positioned at level L5 using an elastic belt. It is important to keep the power connector facing upwards and the logo outwards to correctly define the reference system (Figure 1a)

The test begins with patients seated in a standard chair with their arms on either side of their body. After a signal from the clinician, the subject rises from the chair, walks three meters in a straight line at a speed that is normal for them, turns around an obstacle, and finally returns to the chair and sits down. The software used is BTS G-Studio, which has a specific protocol capable of analyzing the TUG test and automatically generates a TUG report with temporal parameters identifying the duration of the different sub-phases [16]. The mathematical method used to identify each sub-phase is the one described in the study by Salarian et al. [17]. Additionally, a detailed description of the practical operation of BTS G-Studio in iTUG analysis, as compared with an optoelectronic system, is provided in the study by Negrini [18].The test can, therefore, be divided into different phases: the first is that of rising from the chair (sit-to-stand sub-phase), walking for 3 m until reaching an obstacle (walking forward sub-phase), turning around the cone (mid-turning sub-phase), walking three m back towards the chair (return walking sub-phase), and then turning and sitting down on the chair (stand-to-sit sub-phase) without using the assistance of their arms, if possible. The test is concluded when the subject is seated again. The final report of the TUG test shows all the spatiotemporal parameters related to the walk for each sub-phase considered: the sit-to-stand, the steady-state gait, the turning, and the turn-to-sit phases [17]. The parameters supplied automatically by the IMU for each trial are: total time duration, sub-phase durations, mean velocity turning (mid-turning and final turning sub-phases), and the maximum trunk flexion angle and its range of motion during sit-to-stand and stand-to-sit sub-phases (Figure 1b).

Furthermore, an instrumental evaluation of stability was carried out using a baropodometric platform (P-Walk BTS Engineering). The stabilometry test measures the oscillations by evaluating the elliptical area containing 95% of sway points, velocities with closed eyes (CE) and opened eyes (OE), and the length of the excursion of the center of pressure. The test we performed had a duration of 30 s, within which the position of the CoP was recorded during quiet standing [19]. Patients were adequately informed about the procedure; the requirements were to maintain a natural standing position with the arms alongside the body, the feet open at an angle of about 30°, and the heels at a distance of about 3 cm. All tests were performed by the same examiner in order to reduce the inter-operator error and to increase the reproducibility of the test; thus, the subjects were given the same information before each test. For each trial condition (EO and EC), three tests were carried out, for which the median scores are reported. Considering the EO condition, subjects were required to stare at a mark fixed at eye level on a wall 1.5 m away.

### 2.5. Statistical Analysis

The statistical analysis was performed with SPSS software. To verify the normality of the parameters, the Kolmogorov–Smirnov test was used. When the normality assumption was not fulfilled, the median and range (minimum–maximum) were evaluated. The differences between variables were evaluated using the Friedman test for paired samples. The probability level for statistical significance in all tests was set at a *p* < 0.05.

## 3. Results

Forty-eight patients (mean age 71 ± 13.66) were recruited for this study; 4 patients did not complete the rehabilitation program and were excluded from the study; a total of 44 patients (34 female and 10 male, mean age 70 ± 14.02) were evaluated before and after back school treatment.

We observed a global pain reduction in patients with LBP that attended the back-school program. This reduction was also associated with clinical improvement of stability, as shown by the POMA balance score increase. When the postural analysis data were examined, a variation was not registered when considering the opened eyes test; instead, in the closed eyes test a significant reduction of the length of CoP was registered (Table 1).

It is interesting to notice that there was a significant reduction of the total duration of the TUG test, and also of the stand-up and sitting phases (Table 1).

The BS groups showed significant improvement in several instrumental TUG (iTUG) parameters, especially in temporal (duration and velocity) parameters.

The BS treatment significantly reduced the total duration of the task and its sub-phases: the stand-to-sit sub-phase and the sit-to-stand phase, the mean velocity of TUG, and of mid-turning and final turning sub-phases increased at a significant level.

## 4. Discussion

As far as we know, this is the first paper to evaluate not only the pain aspect of lower back syndrome after treatment, but also the functional aspect that is not strictly related to this pathology (i.e., timed up and go evaluation). The TUG test provided in this study is an instrumented TUG. While the TUG test taken by an expert operator using a stopwatch has excellent reliability, accuracy, and precision, this measure is subjective and operator-dependent (i.e., a less experienced clinician could affect the quality of the measure). The use of the stopwatch in the clinical setting has several limitations: (a) the identification of the start time and the end time are not easily detectable by the operator; (b) the evaluation of the TUG time requires a high level of attention by the operator, which could decrease when many trials are required; (c) the quantification of sub-phases is not possible.

The instrumented TUG analysis is of considerable interest, as it evaluates the various sub-phases of the test (chair transition, straight-ahead gait, and 180° turn); this allows a better understanding of movement strategies. Considering, for example, the 180° turn, there is a variability between subjects with different gaits and with **or** without balance impairment. A further variation is introduced for patients using an assistive device, such as a walker.

Therefore, the IMU technology implementations for the iTUG quantification of pre- and post- specific therapies have several benefits, including additional performance parameters, generation of reports, fast assessment, and that the patient does not need to be undressed. In addition to this, it is important to consider the ability for self-administration at home and in a clinical environment. This could provide more details and insights about patient performance [16]. Although other variables could have been derived using the data provided by the wearable sensor, as the purpose of this work was to analyze the TUG, which is an automatic functional clinical test, the analysis focused mainly on the evaluation of the duration of the task included in the test. It is known that lower back pain is associated with functional impairment. In particular, the opportunity to analyze the different phases of this test using an inertial measurement instrument made it possible to assert that back school therapy may improve back function, increasing the promptness to position changes and speeding up movements. The changes observed with iTUG represent the effect of the reduction of LBP on functional ability. As the patients experience pain during the movement, the biomechanical result is a slow movement and a higher TUG time. After treatment, the patients feel better, experience less pain, and can get out of the chair faster. No changes are evidenced as far as postural acquisition is concerned. In maintaining postural control, pain in the lumbar area has a minor effect in terms of functional limitation, and therefore one can expect to have no obvious variations in postural control.

## 5. Conclusions

In conclusion, through the quantitative evaluation of the iTUG test, it is proven that the BS could be considered a promising new rehabilitative treatment for LBP in improving motor functional limitations. Moreover, as the IMU sensor can provide data that might provide many more temporal and kinematic measures after successive elaboration, future development of this study should provide additional data for a more detailed analysis, in order to show more important changes in patients’ movement patterns after the treatment.

## Figures and Tables

**Table 1:** Clinical scale and instrumental evaluation before and after back school cycle.

		T0 (Median ± s.d.)	T1 (Median ± s.d.)	*p*	Chi Quadro	df
POMA Balance		12.88 ± 2.00	13.86 ± 1.92	0.000	17.19	1
NRS		6.11 ± 1.57	4.32 ± 1.99	0.000	33	1
ODI		30.51 ± 13.29	28.72 ± 14.91	0.60	0.273	1
Stabilometria	Area OE (mm^2^)	210.27 ± 1012.07	231.84 ± 1007.86	0.75	0.1	1
	Lenght OE (mm)	115.24 ± 76.58	126.28 ± 99.40	0.15	2.07	1
	Area CE (mm^2^)	446.73 ± 2540.10	591.74 ± 3412.65	0.42	0.64	1
	Length OE (mm)	167 ± 308.20	162.33 ± 221.95	0.02	4.9	1
TUG	Total time (s)	13.37 ± 3.86	11.25 ± 2.16	0.00	19.70	1
	Stand up (s)	1.65 ± 0.37	1.47 ± 0.29	0.02	5.15	1
	Sitting (s)	2.20 ± 0.60	1.99 ± 0.42	0.50	0.44	1
	Rotation velocity (°/s)	77.71 ± 19.80	83.23 ± 22.45	0.04	8.52	1

Legend: POMA = performance-oriented mobility assessment; NRS = numeric rating scale; ODI = Oswestry disability index; OE = opened eyes; CE = closed eyes; TUG = timed up and go; s = second.
